# Low Viral Diversity Limits the Effectiveness of Sequence-Based Transmission Inference for SARS-CoV-2

**DOI:** 10.1128/msphere.00544-22

**Published:** 2023-01-25

**Authors:** Mireille Farjo, Christopher B. Brooke

**Affiliations:** a Department of Microbiology, University of Illinois at Urbana-Champaign, Urbana, Illinois, USA; b Carl R. Woese Institute for Genomic Biology, University of Illinois at Urbana-Champaign, Urbana, Illinois, USA

**Keywords:** SARS-CoV-2, genomic epidemiology, transmission networks

## Abstract

Tracking the spread of infection amongst individuals within and between communities has been a major challenge during viral outbreaks. With the unprecedented scale of viral sequence data collection during the severe acute respiratory syndrome coronavirus 2 (SARS-CoV-2) pandemic, the possibility of using phylogenetics to reconstruct past transmission events has been explored as a more rigorous alternative to traditional contact tracing; however, the reliability of sequence-based inference of transmission networks has yet to be directly evaluated. E. E. Bendall, G. Paz-Bailey, G. A. Santiago, C. A. Porucznik, et al. (mSphere 7:e00400-22, 2022, https://doi.org/10.1128/mSphere.00400-22) evaluate the potential of this technique by applying best practices sequence comparison methods to three geographically distinct cohorts that include known transmission pairs and demonstrate that linked pairs are often indistinguishable from unrelated samples. This study clearly establishes how low viral diversity limits the utility of genomic methods of epidemiological inference for SARS-CoV-2.

## COMMENTARY

The mapping of transmission networks is a powerful tool for understanding pathogen dynamics during an outbreak. While these networks have often been constructed using traditional contact tracing methods (1, 2), phylogenetic techniques can also be used to infer transmission linkages between individuals by identifying samples that map closely together on a phylogeny constructed from community sequences (3, 4). Accounting for the spread of subconsensus variants between infected individuals can also enhance sequence-based transmission analyses (5, 6).

These sequence-based methods have been used as an alternative or a supplement to traditional epidemiological tactics, especially in settings where contact tracing is rendered less effective due to widespread host interactions within large interpersonal networks (7–9). The complementation of traditional methods with genomic epidemiology can therefore yield a more robust approach toward mapping transmission networks. For example, genomic methods have been employed to determine epidemiological factors associated with the sustained circulation of antibiotic-resistant Staphylococcus aureus in regions across the globe (10). During the 2016 Ebola outbreak, phylogenies of sample sequences were constructed to track viral spread between countries (11), and sequencing of dengue virus samples has been used to understand transmission dynamics and identify factors that contribute to increased risk of outbreak (12).

The usefulness of these phylodynamic methods depends in part on the amount of genetic variation present within the local pathogen population, however. If the pathogen in question readily generates and preserves mutations, samples from epidemiologically linked individuals may have differing sequences. If little population diversity exists, sequence homology between samples may not necessarily indicate a transmission linkage. Therefore, not all infectious agents are equally appropriate candidates for sequence-based transmission inference.

During the severe acute respiratory syndrome coronavirus 2 (SARS-CoV-2) pandemic, sequence-based inference has been used to identify superspreading events (13), track global viral transmission (14), and map community infection networks (15). However, due to the relatively low mutation rates of coronaviruses (16) and the low levels of within-host genetic diversity observed during acute infections (17–19), it is possible that the viral diversity generated during intracommunity circulation is inadequate to distinguish true transmission linkages from unlinked samples. Insufficient community sampling may also hinder the reliable inference of transmission networks and decrease the accuracy of these methods, as low sequence availability will decrease the quality of any phylogenetic inference. The dependability of sequence-based inference methods for SARS-CoV-2 must therefore be validated before the technique can be confidently used.

Bendall et al. used SARS-CoV-2 sequence data from households where transmission events between close contacts could be determined with high confidence to determine whether sequences from within a known transmission cluster are more similar to each other than to sequences from the broader community (20). This approach allowed for the evaluation of the accuracy of phylodynamic inference in a scenario in which known transmission linkages were already defined.

Drawing on samples collected from three distinct household transmission studies, the authors constructed phylogenetic trees comprised of SARS-CoV-2 sequences from study participants alongside sequences from the surrounding communities. The amount of community sequence data included on each tree was determined by estimating the overall sampling densities in each study region (New York City, Utah, or Puerto Rico). Though sequences sampled from participants within a household generally grouped together on a phylogenetic tree, these clusters were often interspersed with other (sometimes identical) sequences from the surrounding community that were unlikely to be directly linked by transmission. In a situation where the probable transmission linkages were not already known, this lack of differential clustering would confound efforts to accurately resolve transmission chains. The low levels of SARS-CoV-2 genetic diversity within communities thus hinders the detection of transmission chains from sequence data.

The authors also asked whether including subconsensus genetic variants in sequence comparisons could improve efforts to match linked samples by providing an additional level of genetic diversity. They found that the inclusion of subconsensus variants was not always sufficient to resolve known transmission linkages from a larger pool of community sequences. Therefore, while sequence comparisons could confirm transmission between individuals who were already known to be epidemiologically linked (i.e., household pairs), Bendall et al. show that phylogenetic clustering is not sufficient to confidently determine SARS-CoV-2 transmission linkage in the absence of supplemental contact tracing information.

This study highlights important limitations that should be taken into consideration when reconstructing SARS-CoV-2 transmission networks based on sequence data alone. While the authors note that their results may not translate to congregate settings with higher infection densities, the lack of viral diversity observed in more dispersed communities poses a challenge to epidemiological inference. These findings suggest that sequence data should be used to help confirm likely SARS-CoV-2 transmission events, rather than to identify new ones. Furthermore, the authors demonstrate that phylodynamic methods cannot be applied to all infectious agents with equal effectiveness. The background population diversity and underlying biology of the pathogen of interest must be considered before attempting to draw epidemiological conclusions from sequence data.

## References

[1] Kiss IZ, Green DM, Kao RR. 2005. Disease contact tracing in random and clustered networks. Proc Biol Sci 272:1407–1414. doi:10.1098/rspb.2005.3092. 16006334 PMC1560336

[2] Klinkenberg D, Fraser C, Heesterbeek H. 2006. The effectiveness of contact tracing in emerging epidemics. PLoS One 1:e12. doi:10.1371/journal.pone.0000012. 17183638 PMC1762362

[3] Didelot X, Gardy J, Colijn C. 2014. Bayesian inference of infectious disease transmission from whole-genome sequence data. Mol Biol Evol 31:1869–1879. doi:10.1093/molbev/msu121. 24714079 PMC4069612

[4] Harris SR, Feil EJ, Holden MTG, Quail MA, Nickerson EK, Chantratita N, Gardete S, Tavares A, Day N, Lindsay JA, Edgeworth JD, de Lencastre H, Parkhill J, Peacock SJ, Bentley SD. 2010. Evolution of MRSA during hospital transmission and intercontinental spread. Science 327:469–474. doi:10.1126/science.1182395. 20093474 PMC2821690

[5] Dhar S, Zhang C, Mandoiu I, Bansal MS. 2020. TNet: phylogeny-based inference of disease transmission networks using within-host strain diversity, p 203–216. *In* Cai Z, Mandoiu I, Narasimhan G, Skums P, Guo X (ed), Bioinformatics research and applications. Lecture notes in computer science. Springer International Publishing, Cham, Switzerland. doi:10.1007/978-3-030-57821-3_18.

[6] Wymant C, Hall M, Ratmann O, Bonsall D, Golubchik T, de Cesare M, Gall A, Cornelissen M, Fraser C, STOP-HCV Consortium, The Maela Pneumococcal Collaboration, The BEEHIVE Collaboration. 2018. PHYLOSCANNER: inferring transmission from within- and between-host pathogen genetic diversity. Mol Biol Evol 35:719–733. doi:10.1093/molbev/msx304. 29186559 PMC5850600

[7] Azarian T, Maraqa NF, Cook RL, Johnson JA, Bailey C, Wheeler S, Nolan D, Rathore MH, Morris JG, Jr, Salemi M. 2016. Genomic epidemiology of methicillin-resistant Staphylococcus aureus in a neonatal intensive care unit. PLoS One 11:e0164397. doi:10.1371/journal.pone.0164397. 27732618 PMC5061378

[8] Cairns MD, Preston MD, Lawley TD, Clark TG, Stabler RA, Wren BW. 2015. Genomic epidemiology of a protracted hospital outbreak caused by a toxin A-negative Clostridium difficile sublineage PCR ribotype 017 strain in London, England. J Clin Microbiol 53:3141–3147. doi:10.1128/JCM.00648-15. 26179308 PMC4572532

[9] Séraphin MN, Didelot X, Nolan DJ, May JR, Khan MSR, Murray ER, Salemi M, Morris JG, Lauzardo M. 2018. Genomic investigation of a Mycobacterium tuberculosis outbreak involving prison and community cases in Florida, United States. Am J Trop Med Hyg 99:867–874. doi:10.4269/ajtmh.17-0700. 29987998 PMC6159577

[10] Steinig E, Aglua I, Duchene S, Meehan MT, Yoannes M, Firth C, Jaworski J, Drekore J, Urakoko B, Poka H, Wurr C, Ebos E, Nangen D, Müller E, Mulvey P, Jackson C, Blomfeldt A, Aamot HV, Laman M, Manning L, Earls M, Coleman DC, Greenhill A, Ford R, Stegger M, Syed MA, Jamil B, Monecke S, Ehricht R, Smith S, Pomat W, Horwood P, Tong SYC, McBryde E. 2022. Phylodynamic signatures in the emergence of community-associated MRSA. Proc Natl Acad Sci USA 119:e2204993119. doi:10.1073/pnas.2204993119. 36322765 PMC9659408

[11] Quick J, Loman NJ, Duraffour S, Simpson JT, Severi E, Cowley L, Bore JA, Koundouno R, Dudas G, Mikhail A, Ouédraogo N, Afrough B, Bah A, Baum JHJ, Becker-Ziaja B, Boettcher J-P, Cabeza-Cabrerizo M, Camino-Sánchez Á, Carter LL, Doerrbecker J, Enkirch T, García Dorival IG, Hetzelt N, Hinzmann J, Holm T, Kafetzopoulou LE, Koropogui M, Kosgey A, Kuisma E, Logue CH, Mazzarelli A, Meisel S, Mertens M, Michel J, Ngabo D, Nitzsche K, Pallasch E, Patrono LV, Portmann J, Repits JG, Rickett NY, Sachse A, Singethan K, Vitoriano I, Yemanaberhan RL, Zekeng EG, Racine T, Bello A, Sall AA, Faye O, et al. 2016. Real-time, portable genome sequencing for Ebola surveillance. Nature 530:228–232. doi:10.1038/nature16996. 26840485 PMC4817224

[12] Salje H, Wesolowski A, Brown TS, Kiang MV, Berry IM, Lefrancq N, Fernandez S, Jarman RG, Ruchusatsawat K, Iamsirithaworn S, Vandepitte WP, Suntarattiwong P, Read JM, Klungthong C, Thaisomboonsuk B, Engø-Monsen K, Buckee C, Cauchemez S, Cummings DAT. 2021. Reconstructing unseen transmission events to infer dengue dynamics from viral sequences. Nat Commun 12:1810. doi:10.1038/s41467-021-21888-9. 33753725 PMC7985522

[13] Wang L, Didelot X, Yang J, Wong G, Shi Y, Liu W, Gao GF, Bi Y. 2020. Inference of person-to-person transmission of COVID-19 reveals hidden super-spreading events during the early outbreak phase. Nat Commun 11:5006. doi:10.1038/s41467-020-18836-4. 33024095 PMC7538999

[14] Miller D, Martin MA, Harel N, Tirosh O, Kustin T, Meir M, Sorek N, Gefen-Halevi S, Amit S, Vorontsov O, Shaag A, Wolf D, Peretz A, Shemer-Avni Y, Roif-Kaminsky D, Kopelman NM, Huppert A, Koelle K, Stern A. 2020. Full genome viral sequences inform patterns of SARS-CoV-2 spread into and within Israel. Nat Commun 11:5518. doi:10.1038/s41467-020-19248-0. 33139704 PMC7606475

[15] Aggarwal D, Warne B, Jahun AS, Hamilton WL, Fieldman T, Du Plessis L, Hill V, Blane B, Watkins E, Wright E, Hall G, Ludden C, Myers R, Hosmillo M, Chaudhry Y, Pinckert ML, Georgana I, Izuagbe R, Leek D, Nsonwu O, Hughes GJ, Packer S, Page AJ, Metaxaki M, Fuller S, Weale G, Holgate J, Brown CA, Orton A, Douthwaite J, Rees S, Brown C, Clark R, Jones DR, Kuenzi F, Rankin J, Waddell I, Maxwell P, Matheson N, Abell C, Braithwaite V, Brierley C, Crowcroft J, Dahal A, Faulkner K, Glover M, Goodfellow I, Greatorex J, James L, Lehner P, The Cambridge Covid-19 Testing Centre, University of Cambridge Asymptomatic COVID-19 Screening Programme Consortium, COVID-19 Genomics UK (COG-UK) Consortium, et al. 2022. Genomic epidemiology of SARS-CoV-2 in a UK university identifies dynamics of transmission. Nat Commun 13:751. doi:10.1038/s41467-021-27942-w. 35136068 PMC8826310

[16] Ferron F, Subissi L, Silveira De Morais AT, Le NTT, Sevajol M, Gluais L, Decroly E, Vonrhein C, Bricogne G, Canard B, Imbert I. 2018. Structural and molecular basis of mismatch correction and ribavirin excision from coronavirus RNA. Proc Natl Acad Sci USA 115:E162–E171. doi:10.1073/pnas.1718806115. 29279395 PMC5777078

[17] Braun KM, Moreno GK, Wagner C, Accola MA, Rehrauer WM, Baker DA, Koelle K, O’Connor DH, Bedford T, Friedrich TC, Moncla LH. 2021. Acute SARS-CoV-2 infections harbor limited within-host diversity and transmit via tight transmission bottlenecks. PLoS Pathog 17:e1009849. doi:10.1371/journal.ppat.1009849. 34424945 PMC8412271

[18] Farjo M, Koelle K, Martin MA, Gibson LL, Walden KKO, Rendon G, Fields CJ, Alnaji FG, Gallagher N, Luo CH, Mostafa HH, Manabe YC, Pekosz A, Smith RL, McManus DD, Brooke CB. 2022. Within-host evolutionary dynamics and tissue compartmentalization during acute SARS-CoV-2 infection. bioRxiv. doi:10.1101/2022.06.21.497047. PMC10805032 38174928

[19] Valesano AL, Rumfelt KE, Dimcheff DE, Blair CN, Fitzsimmons WJ, Petrie JG, Martin ET, Lauring AS. 2021. Temporal dynamics of SARS-CoV-2 mutation accumulation within and across infected hosts. PLoS Pathog 17:e1009499. doi:10.1371/journal.ppat.1009499. 33826681 PMC8055005

[20] Bendall EE, Paz-Bailey G, Santiago GA, Porucznik CA, Stanford JB, Stockwell MS, Duque J, Jeddy Z, Veguilla V, Major C, Rivera-Amill V, Rolfes MA, Dawood FS, Lauring AS. 2022. SARS-CoV-2 genomic diversity in households highlights the challenges of sequence-based transmission inference. mSphere 7:e00400-22. doi:10.1128/msphere.00400-22. 36377913 PMC9769559

